# Tumour microbiota structure predicts hypopharyngeal carcinoma recurrence and metastasis

**DOI:** 10.1080/20002297.2022.2146378

**Published:** 2022-11-14

**Authors:** Xiaohui Yuan, Hui‑Ching Lau, Yujie Shen, Qiang Huang, Huiying Huang, Ming Zhang, Lei Tao, Chi-Yao Hsueh, Hongli Gong, Liang Zhou

**Affiliations:** a Department of Otorhinolaryngology Head and Neck Surgery, Eye and ENT Hospital, Fudan University, Shanghai, China; b Shanghai Key Clinical Disciplines of Otorhinolaryngology, Shanghai, China

**Keywords:** Hypopharyngeal carcinoma, microbiota, recurrence, metastasis, 16S rRNA sequencing, disease-free survival

## Abstract

**Objectives:**

The relationship between microbiota and HPSCC recurrence and metastasis remains uncertain. This study aimed to investigate the role of the tumour microbiota in the disease-free survival (DFS) of HPSCC patients.

**Materials and methods:**

Formalin-fixed paraffin-embedded (FFPE) tumour tissues were collected from 103 patients with HPSCC for 16S rRNA sequencing. We analysed the tumour microbiota in HPSCC patients with recurrence/metastasis and nonrecurrence/metastasis. The linear predictor score (LPS) was calculated based on the Cox regression model to assess the risk of recurrence and metastasis. Then, a time-dependent ROC curve was used to evaluate the prognostic power of the LPS.

**Results:**

The phyla *Bacteroidota, Firmicutes* and *Proteobacteria* were the most abundant bacterial taxa in the tumour tissues. *Eubacterium_coprostanoligenes_group* (hazard ratio [HR] = 0.289, 95% confidence interval [CI] 0.137–0.608, *p*= 0.001) and *Prevotella* (HR = 3.744, 95% CI 1.439–9.738, *p*= 0.007) were independent predictors of DFS. The predicting classifier for recurrence and metastasis risk yielded an area under the curve (AUC) of 0.838 at 3 years and 0.860 at 5 years.

**Conclusion:**

Our study demonstrated the relationship between tumour microbiota and recurrence and metastasis in patients with HPSCC.

## Introduction

Hypopharyngeal squamous cell carcinoma (HPSCC) accounts for approximately 3 ~ 5% of head and neck squamous cell carcinomas (HNSCC) [1]. As clinical symptoms in the early stage are atypical, most patients already have advanced stage disease at the time of diagnosis. Currently, multimodality treatment including surgery, radiotherapy and chemoradiotherapy is the most common treatment for advanced HPSCC [2]. Even with active treatment, the prognosis of most patients is dismal, with a 5-year survival rate of less than 50% [3–5]. High rates of recurrence and metastasis are major factors affecting patient survival [6]. Therefore, it is important to identify those HPSCC patients with a high risk of recurrence and metastasis after treatment. Unfortunately, there is no stable prediction model. Clinicopathological characteristics currently used as prognostic indicators in clinical practice lack uniform standards and are insufficient to accurately predict the oncologic outcomes [7,8]. New biomarkers may provide more accurate risk stratification of recurrence and metastasis than clinicopathological characteristics and allow more sensitive disease monitoring.

The human body is a complex ecosystem that hosts tens of thousands of microbial communities. Dysbiosis of the symbiotic microbiota exerts serious consequences on human health. A close association between the microbiota and malignancies has been shown in many cancers [9–12]. The oral microbiota is involved in the development of cancer by stimulating chronic inflammation, inhibiting apoptosis and producing carcinogenic substances [13]. Meanwhile, the role of the microbiota in the prognosis of tumour patients is coming to light. It has been reported that the tumour microbiota is related to prognosis in patients with oesophageal and pancreatic cancers [14,15]. Previous studies demonstrated that HPV infection influences the oral microbiota structure [16,17] and that HPV-positive patients present improved prognosis in HNSCC [18,19], suggesting that the microbiota may become a useful predictor of disease progression and patient outcomes.

However, little is known regarding the association of the microbiota with oncological outcomes in HPSCC. Prognostic biomarkers for HPSCC recurrence and metastasis remain uncertain. To gain insights into the impact of the microbiota on tumour recurrence and metastasis, we collected formalin-fixed paraffin-embedded (FFPE) tumour tissues of HPSCC and divided them into two groups based on the recurrence or metastasis outcomes. Microbial differences between the groups were analysed using 16S rRNA sequencing analysis. Our study found that the HPSCC microbiota affected tumour recurrence and metastasis and that the tumour microbiota can be a predictor of DFS, suggesting the utility of tumour microbiota as a prognostic biomarker in patients with HPSCC.

## Materials and methods

### Study design and participants

This retrospective cohort study was performed at the Department of Otorhinolaryngology at the Eye and ENT Hospital of Fudan University. The protocol of this study was approved by the Ethics Committee of the Eye and ENT Hospital, and informed consent was obtained before screening.

A total of 112 patients who accepted hypopharyngectomy with curative intent from January 2015 to January 2018 were enrolled in this study. Subsequent follow-up was performed every three months for the first year and every six months or annually thereafter, including outpatient follow-up and telephone follow-up. Follow-up continued until recurrence, metastasis, death or the last follow-up date (December 2021). Patients were divided into two groups: nonrecurrence/metastasis (NR) and recurrence/metastasis (RC). Recurrence/metastasis was referred to as tumour recurrence (local or regional) and distant metastasis detected by imaging or pathological examinations. Disease-free survival (DFS) was defined as survival time from the end of treatment to recurrence (local, regional), distant metastasis or death.

The inclusion criteria for this study were as follows: (1) pathologically confirmed squamous cell carcinoma of the hypopharyngeal region; (2) written informed consent obtained before participation; and (3) complete clinical, pathological and follow-up information available. The exclusion criteria were as follows: (1) use of antibiotics or steroids within the past 3 months and (2) multiple primary malignant tumours.

### Sample collection, DNA extraction and HPV DNA detection

Archived FFPE tumour tissues were collected. Sections (10 µm thick) were prepared from FFPE tumour tissue samples. Total genomic DNA was extracted using the GeneRead DNA FFPE Kit (Qiagen, Germany) following the manufacturer’s protocol. DNA quality and concentration were verified by Nanodrop 2000 and 1% agarose gel electrophoresis. Detection of HPV DNA was performed by quantitative real-time PCR with HPV Genotyping Real-time PCR kit (Hybribio Biotech, China) and an ABI 7500 real-time PCR system (Applied Biosystems, USA). Clinicopathological characteristics were collected from medical records and pathology results. Pathological TNM staging was evaluated based on the eighth edition of the American Joint Committee on Cancer (AJCC).

### DNA amplification and 16S rRNA sequencing

The extracted DNA was used for PCR with Tks Gflex DNA Polymerase. V3-V4 variable regions of 16S rRNA genes were amplified using universal primers 343 F (5’-TACGGRAGGCAGCAG-3’) and 798 R (5’-AGGGTATCTAATCCT-3’). The PCR products were quality checked using gel electrophoresis and purified with AMPure XP beads (Beckman Coulter, USA). Then, the products were quantified using a Qubit dsDNA assay kit. Purified amplicons were pooled in equal amounts and then sequenced on an Illumina NovaSeq6000 platform (Illumina Inc., USA) with two paired-end read cycles of 250 bases each.

Raw sequencing data were saved in FASTQ format. Paired-end reads were processed to cut off the adapter sequence using cutadapt software. Amplicon sequence variants (ASVs) were obtained after quality filtering, denoising, merging and removing of the chimaera reads using DADA2 [20] in the QIIME2 [21] platform. Then, the q2-feature classifier was used to annotate all ASVs against Silva database version 138.

### Bioinformatic analysis

To measure the microbial diversity, alpha diversity and beta diversity were calculated using QIIME2 and R (version 4.1.1). Alpha diversity was used to evaluate species evenness within samples through the Shannon index and Simpson index. The Wilcoxon rank-sum test was used to examine alpha diversity differences between the NR and RC groups. Beta diversity analysis assessed the similarity and dissimilarity of the microbial community between the NR and RC groups. Principal co-ordinates analysis (PCoA) based on the Bray-Curtis distance matrix and unweighted UniFrac distance matrix combined with Adonis analysis was used to assess the difference between groups. Linear discriminant analysis (LDA) coupled with effect size measurements (LEfSe) was conducted to detect bacterial taxa with significantly differential abundance between the two groups.

### Statistical analysis

Pearson’s chi-square test or Fisher’s exact test was used to analyse comparisons between categorical variables. Student’s t test was used for normally distributed variables, and the Mann–Whitney U test was used for nonnormally distributed variables. The cut-off value of the continuity variable was calculated using X-tile software. The survival curves were generated using Kaplan–Meier analysis. The difference in survival curves between groups was tested using the log-rank test. Multivariate Cox regression models were used to identify the statistical power of variables on DFS. Using the independent modules in the multivariate Cox regression models, the linear predictor score (LPS), which sums the result of multiplying mean-centred covariate values and their corresponding β coefficients, was calculated as an indicator to assess the risk of recurrence. The calculation formula was as follows: ΣX_i_*β. The R package ‘survival ROC’ was used to construct a survival ROC curve and calculate the area under the curve (AUC). Two-sided P values < 0.05 were considered statistically significant. All analyses were conducted using SPSS (version 25.0), GraphPad Prism (Version 8.0.2) and R (Version 4.1.1) software.

## Results

### Patient characteristics and sequencing data

**Figure 1.**
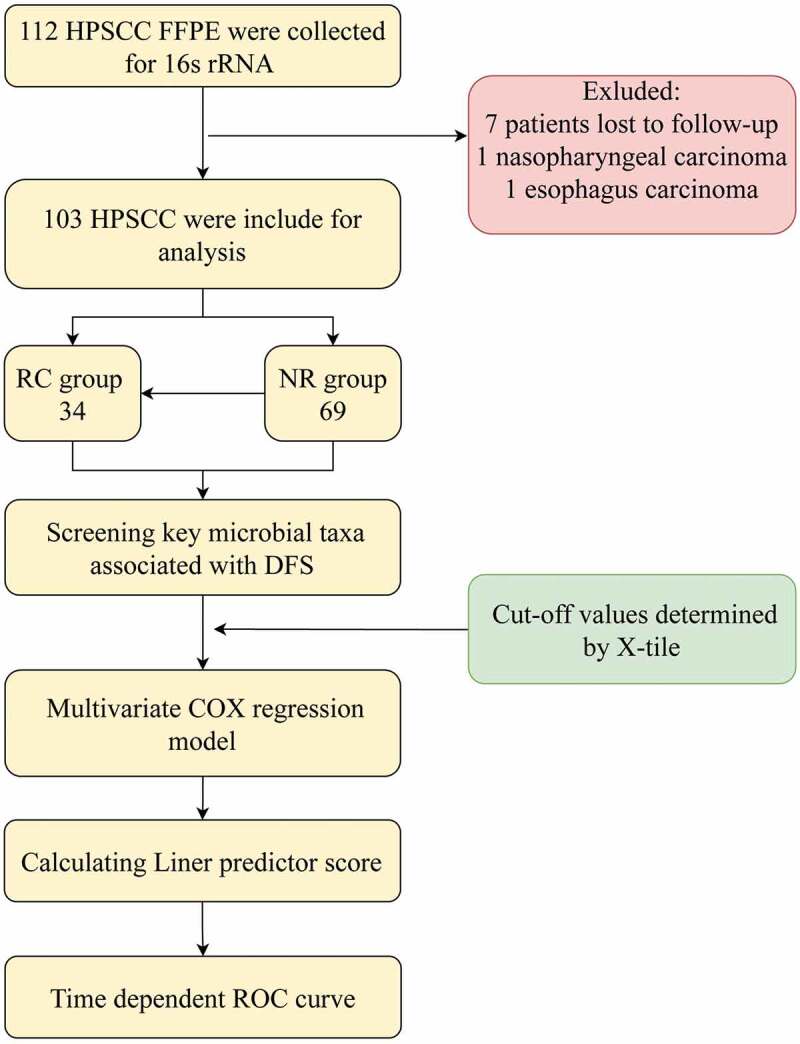
Flow chart of the current study procedure. HPSCC, hypopharyngeal squamous cell carcinoma; FFPE, formalin-fixed paraffin-embedded; RC, recurrence/metastasis; NC, nonrecurrence/metastasis; DFS, disease-free survival.

One hundred and three patients were eligible for sequencing and analysis upon critical review. Nine patients were excluded, including seven patients lost to follow-up and two patients diagnosed with multiple primary malignant tumours (Figure 1). The median follow-up was 56 months. Among 34 patients who experienced recurrences, 17 had local recurrence, 8 had regional recurrence, 2 had local-regional recurrence and 7 had distant metastases. Clinical data are detailed in Table 1.

**Table 1.** Clinicopathological characteristics of HPSCC patients enrolled in this study.

Variables	Total Patients (%)	NR group (n = 69)	RC group (n = 34)	*P* value
Age (years)				0.537
≤60	56 (54.4%)	36 (52.2%)	20 (58.8%)	
>60	47 (45.6%)	33 (47.8%)	14 (41.2%)	
Sex				0.481
Male	102 (99.0%)	68 (98.6%)	34 (100.0%)	
Female	1 (1.0%)	1 (1.4%)	0	
HPV status				0.019
Positive	15 (14.6%)	14 (20.3%)	1 (2.9%)	
Negative	88 (85.4%)	55 (79.7%)	33 (97.1%)	
Smoking history				0.244
Yes	77 (74.8%)	54 (78.3%)	23 (67.6%)	
No	26 (25.2%)	15 (21.7%)	11 (32.4%)	
Drinking history				0.698
Yes	67 (65.0%)	44 (63.8%)	23 (67.6%)	
No	36 (35.0%)	25 (36.2%)	11 (32.4%)	
Tumor Subregion				0.344
Pyriform sinus	88 (85.4%)	61 (88.4%)	27 (79.4%)	
Postcricoid region	7 (6.8%)	3 (4.3%)	4 (11.8%)	
Posterior pharyngeal	8 (7.8%)	5 (7.2%)	3 (8.8%)	
T staging^a^				0.102
T1	7 (6.8%)	6 (8.7%)	1 (2.9%)	
T2	36 (35.0%)	25 (36.2%)	11 (32.4%)	
T3	27 (26.2%)	21 (30.4%)	6 (17.6%)	
T4	33 (32.0%)	17 (24.6%)	16 (47.1%)	
N staging^a^				0.561
N0	14 (13.6%)	11 (15.9%)	3 (8.8%)	
N1	23 (22.3%)	17 (24.6%)	6 (17.6%)	
N2	45 (43.7%)	28 (40.6%)	17 (50.0%)	
N3	21 (20.4%)	13 (18.8%)	8 (23.5%)	
TNM staging^a^				0.316
Stage I–II	2 (1.9%)	2 (2.9%)	0	
Stage III–IV	101 (98.1%)	67 (97.1%)	34 (100%)	
Lymphovascular invasion				0.132
Yes	9 (8.7%)	4 (5.8%)	5 (14.7%)	
No	94 (91.3%)	65 (94.2%)	29 (85.3%)	
Tumor differentiation				0.977
Well and moderately	88 (85.4%)	59 (85.5%)	29 (85.3%)	
Poor	15 (14.6%)	10 (14.5%)	5 (14.7%)	

^a^TNM staging was based on the eighth edition of the AJCC

The data volume of raw reads of each sample was between 73,111 and 81,981. After quality control, the data volume of clean tags of each sample was between 19,967 and 61,893. Chimera reads were removed, and the number of valid tags finally used for analysis ranged from 19,565 to 59,833. Each sample contained between 128 and 1437 ASVs.

### Tumour microbiota diversity and composition

**Figure 2.**
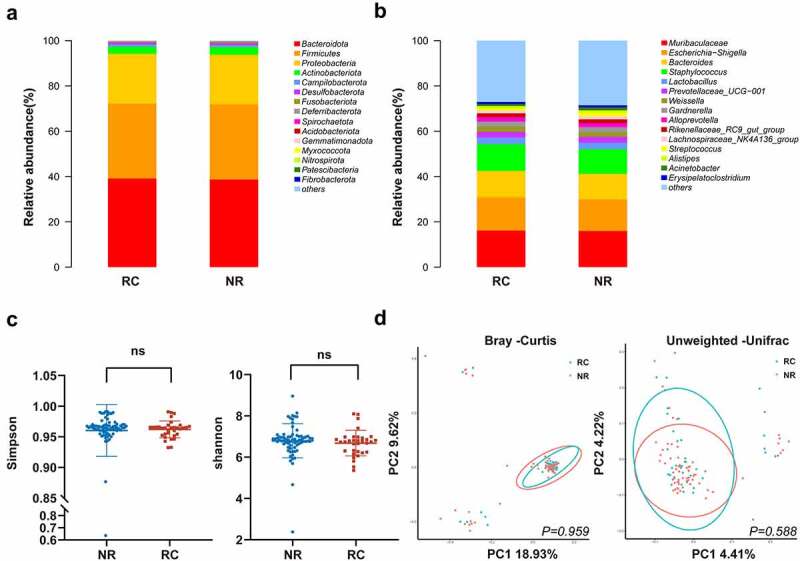
The microbial community structure in tumour tissues showed homogeneity between the nonrecurrence/metastasis (NR) and recurrence/metastasis (RC) groups. (A) Average composition of the bacterial community at the phylum level. (B) Average composition of the bacterial community at the genus level. (C) Alpha diversity values were determined as Simpson (P = 0.286) and Shannon (P = 0.183) indices. Error bars represent the standard deviation (SD) of diversity scores. (D) PCoA plots for beta diversity were based on Bray-Cutis and Unweighted UniFrac distance matrix. Circles represent 95% confidence intervals (CIs).

We first assessed the general tumour microbiota profile of HPSCC tissue samples. No significant difference in the dominant bacterial proportions was found between the RC and NR groups (Figure 2A and 2B). The results showed that the top 5 bacteria at the phylum level were *Bacteroidota, Firmicutes, Proteobacteria, Actinobacteriota* and *Campilobacterota. Bacteroidota, Firmicutes* and *Proteobacteria* accounted for more than 90%. The five most abundant taxa at the genus level were *Muribaculaceae, Escherichia-Shigella, Bacteroides, Staphylococcus* and *Lactobacillus*. Then, we explored the tumour microbial diversity between the NR and RC groups to reveal the general microbial community structure. We analysed alpha diversity to determine the richness and evenness of each tumour microbiota sample. There was no significant difference in alpha diversity between the two groups (Figure 2C). We compared the microbiota distributions and compositions among the two groups by calculating beta diversity using different distance matrices. As expected, no difference in tumour microbiota distribution or composition was found between the NR and RC groups (Figure 2D). These data suggest that the microbial community diversity and structure were very similar between the two groups.

### Correlation between tumour microbiota and HPV status

**Figure 3.**
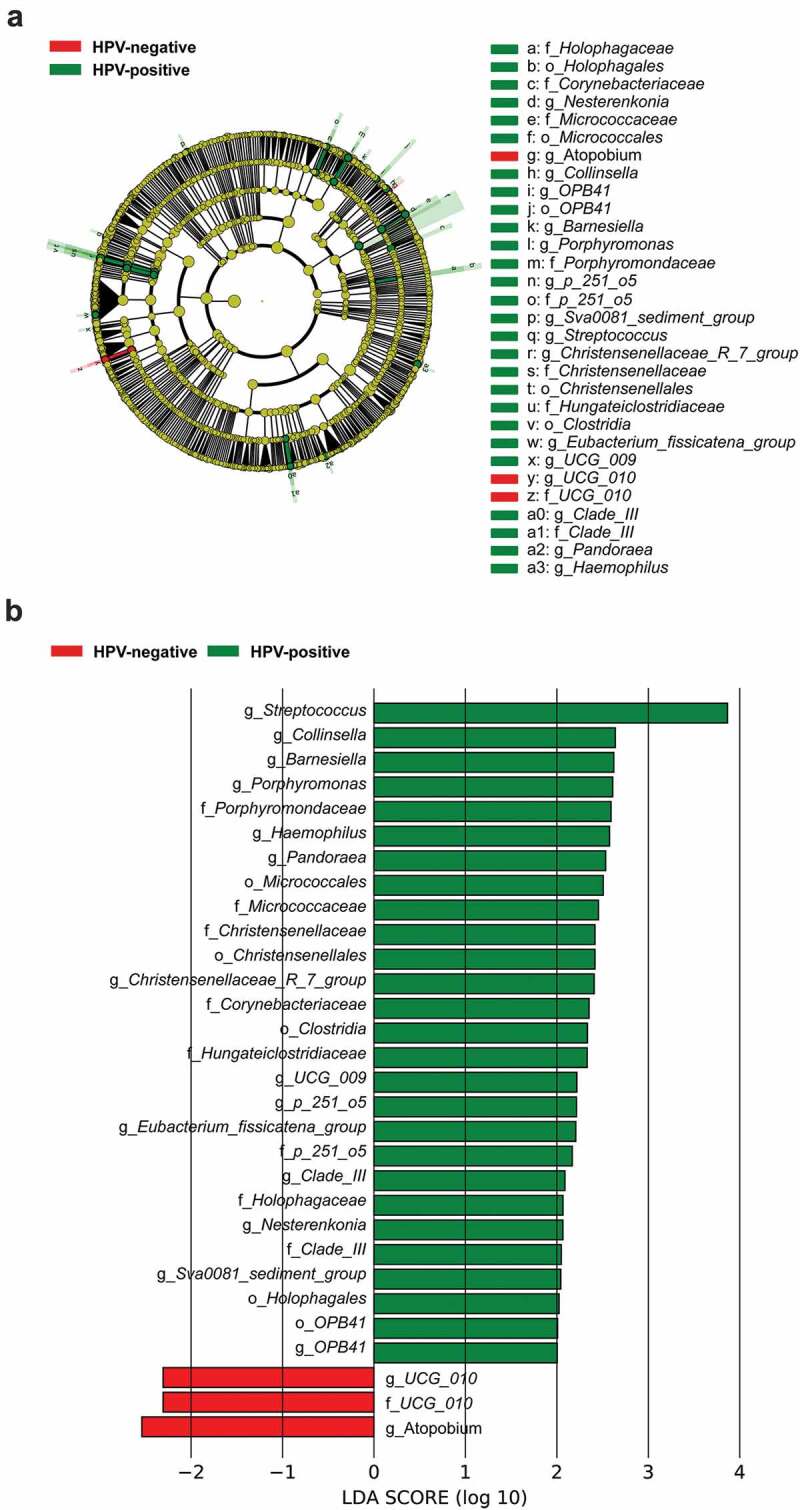
Microbial differences between HPV-positive and HPV-negative groups. LEfSe analysis was used to identify differentially abundant taxa between groups. (A) Cladogram; (B) histogram.

To identify the extent to which HPV infection impacts tumour microbiota, LEfSe was used to evaluate differences between HPV-positive and HPV-negative groups. The HPV-positive tumours exhibited an enrichment of *Porphyromondaceae, Micrococcaceae, Christensenellaceae, Corynebacteriaceae* and *Hungateiclostridiaceae* at the family level, as well as *Streptococcus, Collinsella, Barnesiella, Porphyromonas* and *Haemophilus* at the genus level, indicating that HPV infection altered the tumour microbial communities (Figure 3A and 3B). Notably, we found a remarkable increase in *Streptococcus* abundance in the HPV-positive group. PICRUSTS analysis was used to predict the gene functions of the tumour microbiota and to reveal changes in microbial functional pathways across different groups. Transporter-, microbial metabolism- and transcription factor-related pathways were enriched in the microbiota of the HPV-positive group (Fig S1A). Transporter-related pathways included multiple sugar transport and polar amino acid transport. Microbial metabolism-related pathways included glycolysis/gluconeogenesis, pentose phosphate pathway purine metabolism and glycine, serine and threonine metabolism. These results suggest that HPV infection has an impact on microbial metabolism and the way microbiota interacts with hypopharyngeal environment, which may be the cause of the changes in microbiota.

### Identification of key microbial taxa correlated with HPSCC recurrence and metastasis

**Figure 4.**
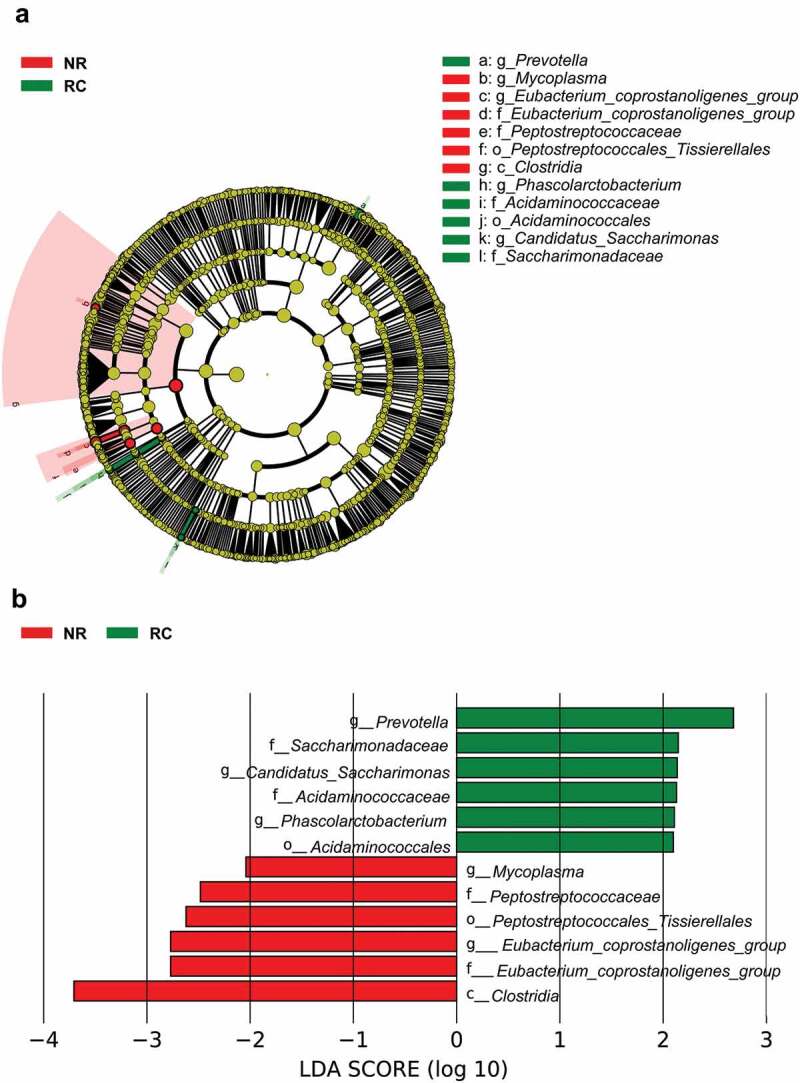
Identification of key microbial taxa correlated with HPSCC recurrence. LEfSe analysis was used to identify differentially abundant taxa between the NR and RC groups. (A) Cladogram; (B) histogram. The genus taxa ranked in the top 30 were selected according to their relative abundance.

**Figure 5.**
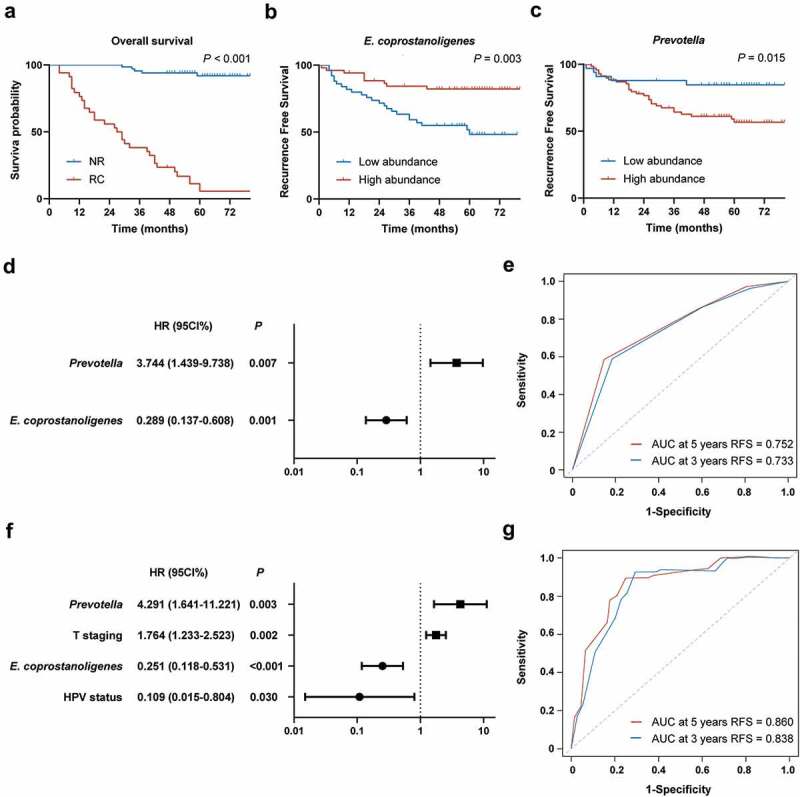
Tumour microbiota were associated with DFS and were predictive of recurrence. (A) Kaplan–Meier curve of overall survival (OS) probabilities between the NR and RC groups. (B to C) Kaplan–Meier curve of DFS probabilities based on the abundance of *Eubacterium_coprostanoligenes_group* and *Prevotella*. (D) Multivariate Cox regression model of microbial variables. Log10 scale is used for the X-axis. (E) Time-dependent ROC curve based on linear predictor score (LPS) from *Eubacterium_coprostanoligenes_group* and *Prevotella*. (F) Multivariate Cox regression model of microbial and clinicopathological variables. Log10 is used for the X-axis. (G) Time-dependent ROC curve based on LPS from microbial and clinicopathological variables. HR, hazard ratio; CI, confidence interval; AUC, area under the curve; *E. coprostanoligenes, Eubacterium_coprostanoligenes_group.*

Considering the decreasing overall survival (OS) in the RC group (Figure 5A), we next sought to explore whether there were marked differences in microbial communities between the NR and RC groups. We focused on the predominance of microbial communities in each group and used LEfSe to reveal the differences in microbial communities between the two groups. We detected a higher abundance of *Prevotella, Candidatus_Saccharimonas* and *Phascolarctobacterium* at the genus level in the RC group (Figure 4). In contrast, the NR group exhibited a predominance of *Mycoplasma* and *Eubacterium_coprostanoligenes_group* at the genus level (Figure 4). Then, the genera ranked in the top 30 were selected according to their relative abundance. After screening, *Prevotella* and *Eubacterium_coprostanoligenes_group* were subjected to survival analysis to further identify key microbial taxa associated with DFS. We then stratified the entire cohort of patients into high abundance and low abundance groups based on the optimal cut-off values of relative abundance determined by X-tile (Figure S1B and S1C). Kaplan–Meier survival analysis revealed a significantly better prognosis in patients with a higher abundance of *Eubacterium_coprostanoligenes_group* (Figure 5B) and alower abundance of *Prevotella* (Figure 5C). Multivariate Cox regression models further revealed that these two taxa were independent predictors of DFS: *Eubacterium_coprostanoligenes_group* (hazard ratio [HR] = 0.289, 95% confidence interval [CI] 0.137–0.608, *p*= 0.001) and *Prevotella* (HR = 3.744, 95% CI1.439–9.738, *p*= 0.007) (Figure 5D). These findings imply that the presence and abundance of *Eubacterium_coprostanoligenes_group* and *Prevotella* may influence and predict recurrence in HPSCC patients.

### Establishment of a predicting classifier for recurrence and metastasis risk

We next used *Eubacterium_coprostanoligenes_group* and *Prevotella* to construct a predicting classifier for estimating recurrence risk in HPSCC patients. First, we calculated the LPS of the two taxa for each patient based on the β coefficients from the Cox regression model (Table 2). Then, a time-dependent ROC curve was developed to evaluate the prognostic power of LPS (Figure 5E). The AUC of the time-dependent ROC was 0.733 at 3 years and 0.752 at 5 years.

**Table 2.** Multivariate cox regression models for DFS.

Variables	β coefficients	SE^a^	Wald^b^	*P* value
Multivariate Cox regression model of microbial variables				
*Eubacterium_coprostanoligenes_group*	−1.242	0.379	10.71	0.001
*Prevotella*	1.32	0.488	7.326	0.007
Multivariate Cox regression model of microbial and clinicopathological variables				
T staging	0.568	0.183	9.653	0.002
HPV status	−2.214	1.019	4.726	0.030
*Eubacterium_coprostanoligenes_group*	−1.384	0.383	13.051	<0.001
*Prevotella*	1.457	0.490	8.820	0.003

^a^SE, standard error; ^b^Wald, Wald Chi-square

Next, clinicopathological variables were evaluated. Univariate analysis showed that HPV status, tumour subregion and T stage were related to the risk of recurrence (Fig. S2). Multivariate Cox regression models proved that HPV status (HR = 0.123, 95% CI 0.017–0.902, *p*= 0.039) and T stage (HR = 1.648, 95% CI 1.135–2.393, *p* = 0.009) were independent predictors of DFS. The LPS of clinicopathological variables showed an AUC of 0.691 at 3 years and 0.683 at 5 years (Fig. S3).

For the more powerful predicting classifier, using two selected microbial taxa and three clinicopathological variables, subsequent multivariate analysis revealed that *Eubacterium_coprostanoligenes_group* (HR = 0.251, 95% CI 0.118–0.531, *p*< 0.001), *Prevotella* (HR = 4.291, 95% CI 1.641–11.221, *p*= 0.003), HPV status (HR = 0.109, 95% CI 0.015–0.804, *p*= 0.030) and T staging (HR = 1.764, 95% CI 1.233–2.523, *p*= 0.002) were independent risk factors (Figure 5F). After calculating the LPS values of the four independent modules and developing a time-dependent ROC curve, the AUC was 0.838 at 3 years and 0.860 at 5 years, implying good performance of the predicting classifier (Figure 5G). For the final model, the sensitivity and specificity at the optimal cutoff value have been calculated. The sensitivity and specificity were 92.7% and 70.7% respectively at 3 years, 89.5% and 75.1% respectively at 5 years.

### Discussion

Our study is the first to delineate the relationship between tumour microbiota and tumour recurrence and metastasis of HPSCC. Phyla *Bacteroidota, Firmicutes* and *Proteobacteria* were the most abundant bacterial taxa in HPSCC tumour tissues. Through comprehensive survival analysis, we found associations between the abundance of *Prevotella* and *Eubacterium_coprostanoligenes_group* and oncological outcomes in HPSCC. A higher abundance of *Prevotella* and a lower abundance of *Eubacterium_coprostanoligenes_group* are indicators of higher rates of recurrence and metastasis. Furthermore, we constructed for the first time a prognostic model based on specific microbes and clinical information. LPS computed from the generated model shows high predictive power.

Previous surveys have reported similar detection of major microbial populations in HNSCC [22,23]. However, the relationship between microbiota and recurrence and metastasis of HPSCC is unclear. Here, no significant difference was observed in microbial diversity between NR and RC cohorts, suggesting that microbiota dysbiosis plays a stable role in tumour development [24]. In addition, few studies have investigated the relationship between HPV and microbial communities in the hypopharynx. In our study, the HPV-positive cohort was proven to have an increased abundance of specific microbes, particularly *Streptococcus*, consistent with other studies [25,26]. *Streptococcus* promotes HPV infection by expressing furin-like peptidases and is involved in ethanol metabolism, resulting in carcinogenesis [25,26]. According to our results, patients with different abundances of key taxa presented different prognoses, indicating that the abundances of the two screened genera enable better prediction of recurrence in HPSCC patients than clinical variables, leading to the conclusion that microbiota examination may be an encouraging approach for clinical application.

**Figure 6.**
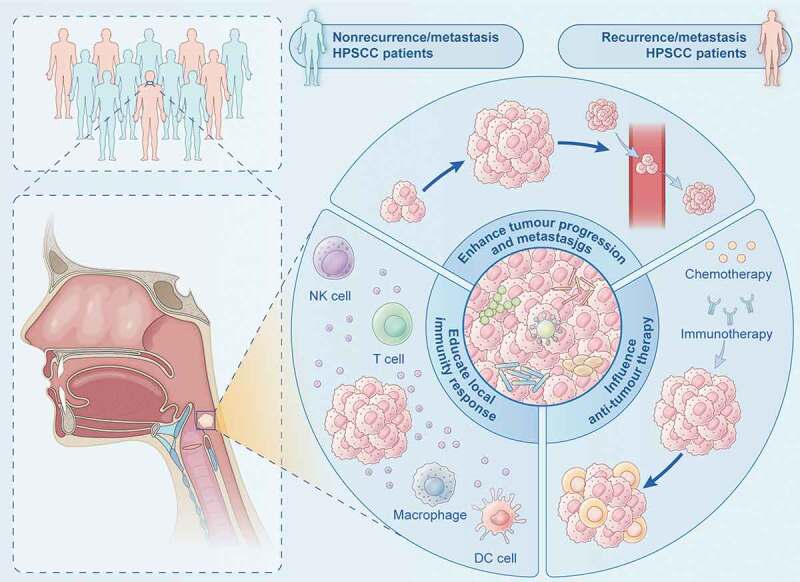
Schematic diagram of the association between intratumor microbiota and oncological outcomes of HPSCC. Microbiota may affect oncological outcomes by enhancing tumour progression and metastasis, educating local immunity response and influencing anti-tumour therapy.

In recent years, increasing researches have confirmed the presence of intratumor microbiota, providing biological basis of the application of intratumor microbiota as a promising prognostic biomarker [27]. Microbiome studies of multiple tumours have demonstrated the microbiota-associated oncological outcomes [14,28]. Although the exact mechanism has not been fully elucidated, tumour microbiota may affect oncological outcomes via (1) enhancing tumour progression and metastasis; (2) educating local immunity response; (3) influencing anti-tumour therapy (Figure 6). Intratumor microbiota can affect tumour progression and metastasis through inducing DNA damage and driving oncogenic pathways [27], which are associated with poor prognosis. Microbiota in tumour can also generate an immune suppressive microenvironment by recruiting immunosuppressive cells, leading to tumour immune escape [29,30]. For example, *Streptococcus gallolyticus* can inhibit T cells by recruiting myeloid-derived suppressor cells (MDSCs), tumour-associated macrophages (TAMs) and dendritic cells (DCs) [31]. The microbial community affects response to anti-tumour therapy. Intratumor microbiota may alter the expression of checkpoint proteins, affecting the efficacy of immunotherapy. It is reported that patients responsive to anti-PD-L1 therapy had high level of *Fusobacterium nucleatum* [32]. Further research found that *Fusobacterium nucleatum* activated STING signaling and accumulated IFN-γ+ CD8+ tumour-infiltrating lymphocytes (TILs) and therefore induced PD-L1 expression, enhancing tumour sensitivity to PD-L1 blockade. Other research reported that *Fusobacterium nucleatum* altered cancer chemotherapeutic response by activating autophagy-related pathways [33]. Intratumor microbiota can also influence the effect of chemotherapy by metabolizing drugs into their inactive forms [27].

An increased abundance of *Prevotella* in the RC group was observed, suggesting that *Prevotella* is intimately associated with the DFS outcomes of patients. The survival analysis performed with patients stratified by the abundance of *Prevotella* showed the prognostic significance of *Prevotella* and its value in predicting the outcomes of patients with HPSCC. *Prevotella*, as a gram-negative bacterium, is part of the normal flora in the oral cavity, pharynx and gut [34]. Similarly, other research has found that the increased abundance of *Prevotella* was associated with poorer prognosis in esophageal cancer [15]. Previous studies have identified the correlations between *Prevotella* and cancers, including laryngeal [35], oral [36], esophageal [37] and colorectal [38] cancers. *Prevotella* was detected both in primary and liver metastatic tumours [39], demonstrating its stability during metastasis. Although the exact molecular mechanism by which *Prevotella* promotes tumorigenesis and progression and influences oncologic outcomes remains unclear, mounting evidence suggests biological plausibility of *Prevotella* for predicting patient prognosis. Recent studies have shown that *Prevotella* affect the anti-tumour immune response. Some species of *Prevotella* such as *Prevotella nigrescens* and *Prevotella copri* have been shown to promote Th17 cell development and enhance the expression of pro-inflammatory cytokines [40,41]. The finding of a correlation between *Prevotella* and the cytokines IL-5, IL-9 and IL-17A in the tumour microenvironment in colorectal cancer confirms a bidirectional interference between the host immune response and microbiota [42]. In addition, *Prevotella* can affect the response to chemotherapy treatment, thus affecting patient prognosis [43].

According to our study, patients with a higher abundance of *Eubacterium_coprostanoligenes_group* presented better DFS. *Eubacterium_coprostanoligenes_group* was thus concluded to be a protective factor for tumour recurrence. As a group of anaerobic gram-positive bacteria, *Eubacterium_coprostanoligenes_group* is able to convert cholesterol to unabsorbable coprostanol, regulating cholesterol levels. Cholesterol is a key component of the cell membrane and is necessary for the rapid proliferation of cancer cells [44]. Cholesterol metabolism plays a role in the proliferation, migration and metastasis of tumour cells [45,46]. A decrease in cholesterol levels in the local microenvironment can inhibit tumour growth and metastasis. A previous study showed that elevated cholesterol levels were related to tumour recurrence in breast cancer [47]. The primary metabolism of cholesterol, 27-hydroxycholesterol (27HC), promotes metastasis in breast cancer by interacting with γδ-T cells and polymorphonuclear neutrophils [47]. The use of statins reduced the risk of recurrence among breast cancer patients [47]. A national study in Denmark also reported that the use of statins was able to reduce cancer-related mortality [48]. In addition, cholesterol metabolites affect the immune microenvironment by enriching immunosuppressive cells, inhibiting immune effector cells and inhibiting antigen presentation [44]. Targeting cholesterol metabolism has provided novel insights for cancer treatment. Therefore, we speculate that *Eubacterium_coprostanoligenes_group* at the primary tumour site exerts effects on cancer metastasis by participating in the local cholesterol metabolism. Future studies should proceed to illuminate the exact relationship between *Eubacterium_coprostanoligenes_group* and cholesterol in cancer metastasis.

Several limitations in our study need to be acknowledged. First, our study is retrospective in nature, and further prospective studies are needed to validate the results. Given that our model lacks external validation, more tumour samples are required to confirm these findings. Moreover, we used relative abundance based on 16S rRNA sequencing data to describe the microbial distribution, which may require validation of absolute abundance. As the understanding of tumour advances, the risk of tumour development and recurrence can be effectively reduced by controlling risk factors such as smoking, alcohol consumption, poor dietary habits and overweight. The impact of post-operative life style of patients on disease recurrence was not considered, which may limit the strength of our conclusion. In addition, the 16S rRNA sequencing technique, which analyses the V3-V4 region of bacterial 16S rRNA gene, could only support bacterial identification at the genus level and is limited in analysis at the specie level.

In conclusion, we uncovered a role for microbiota in recurrence and metastasis in HPSCC for the first time and suggest that tumour microbiome sequencing can be used for patient recurrence and metastasis risk stratification. New opportunities to improve the prognosis of HPSCC patients by using microbial interventions may be found in the future.

## Supplementary Material

Supplemental Material

## Data Availability

All raw reads have been deposited in the NCBI Sequence Read Archive (SRA) under accession number PRJNA833656 (https://www.ncbi.nlm.nih.gov/).
