# Supplementary material for: Tumour microbiota structure predicts hypopharyngeal carcinoma recurrence and metastasis
Source: J Oral Microbiol. 2022 Nov 14;15(1):2146378. doi: 10.1080/20002297.2022.2146378 (PMC9668281; doi:10.1080/20002297.2022.2146378)
Supplement: Supplemental Material [file ZJOM_A_2146378_SM4453.docx]

**Supplementary materials**

**
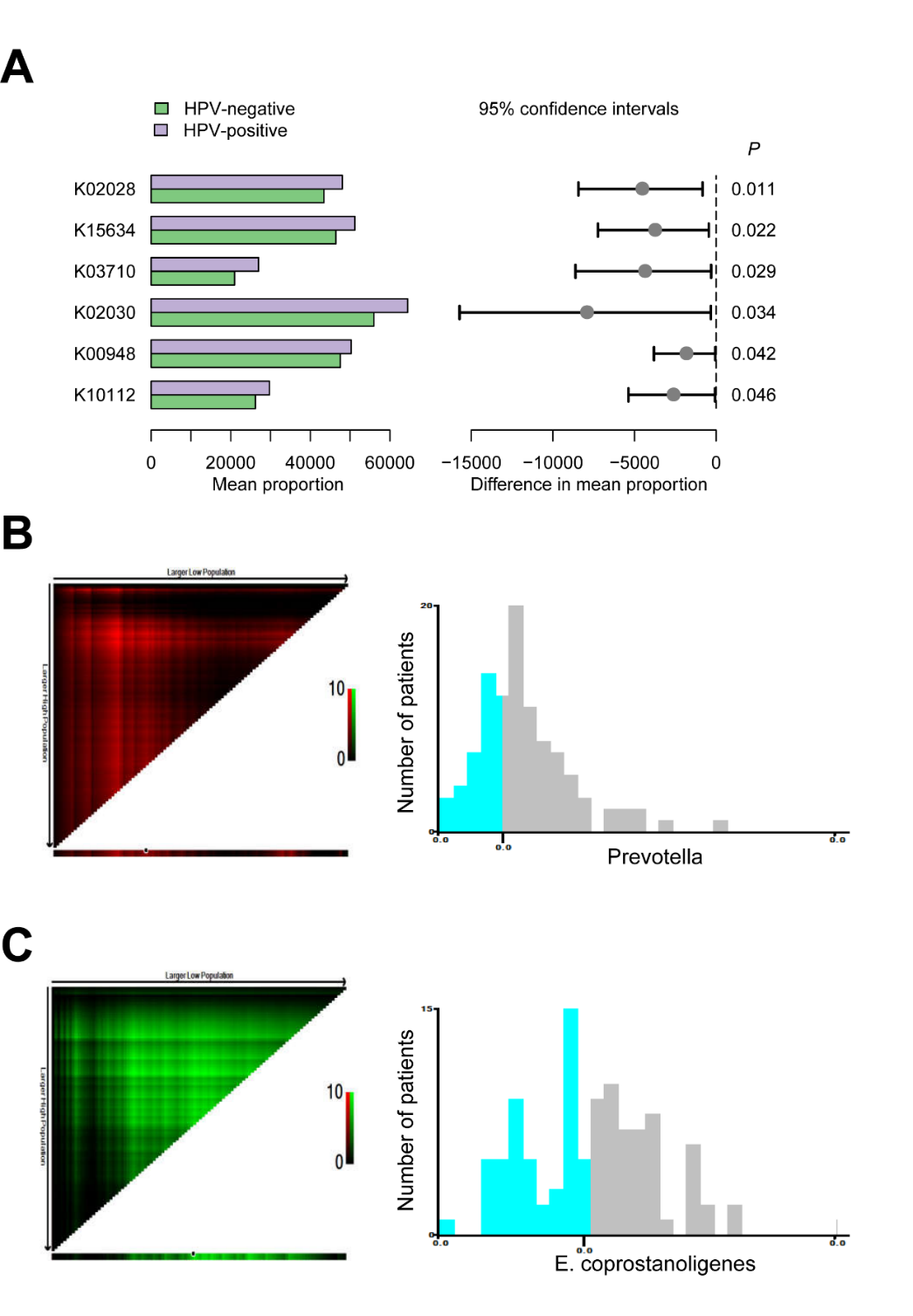
**

Fig. S1. A. Extended error bar for the differences in gene functions as determined by the KEGG Orthology (KO) database. (B-C) X-tile analysis for the determination of cut-offs for *Eubacterium_coprostanoligenes_group* and *Prevotella* abundance.


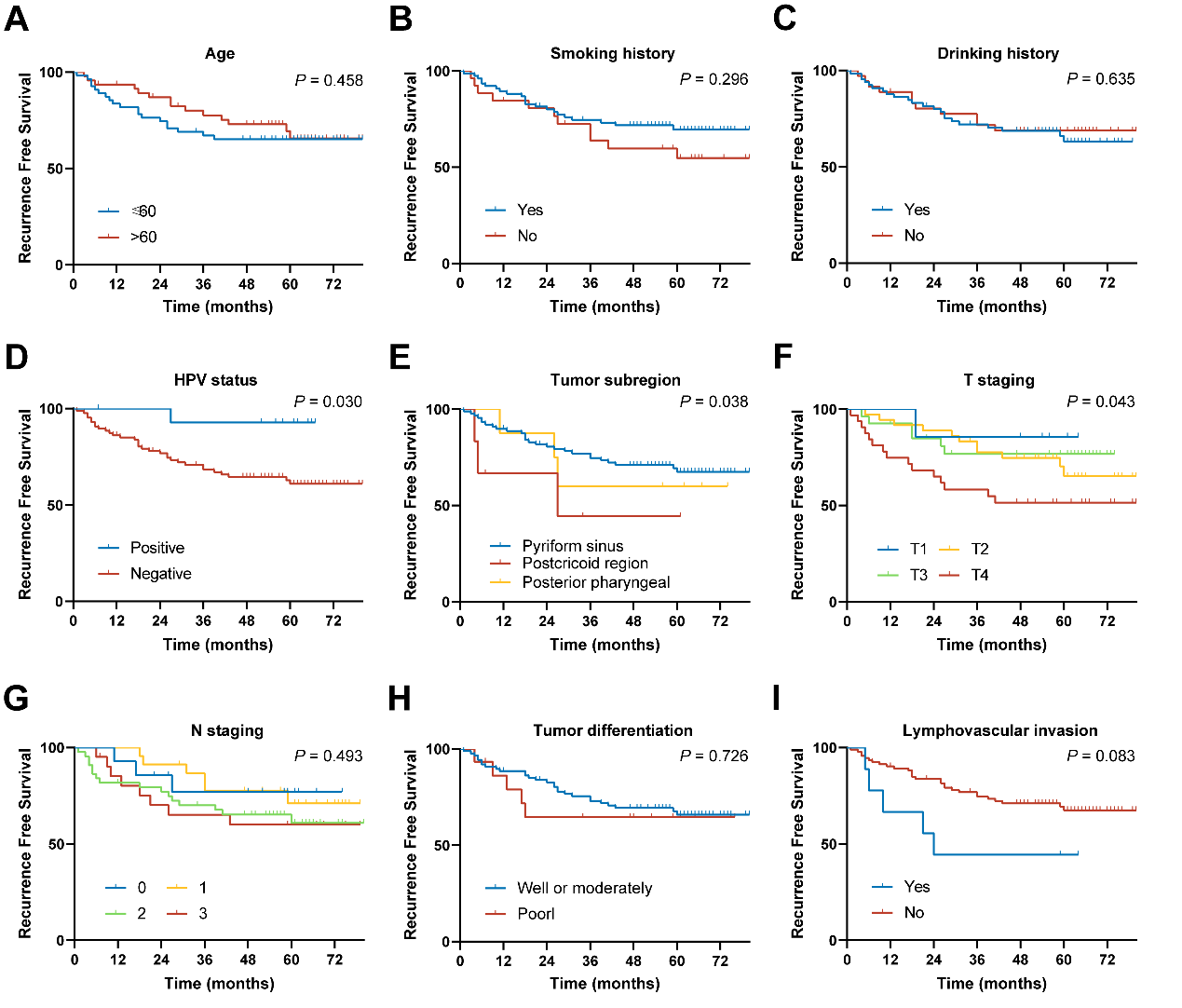


Fig. S2. Univariate analysis of clinicopathological variables using Kaplan–Meier curves. Log-rank analyses were used to determine differences between groups.


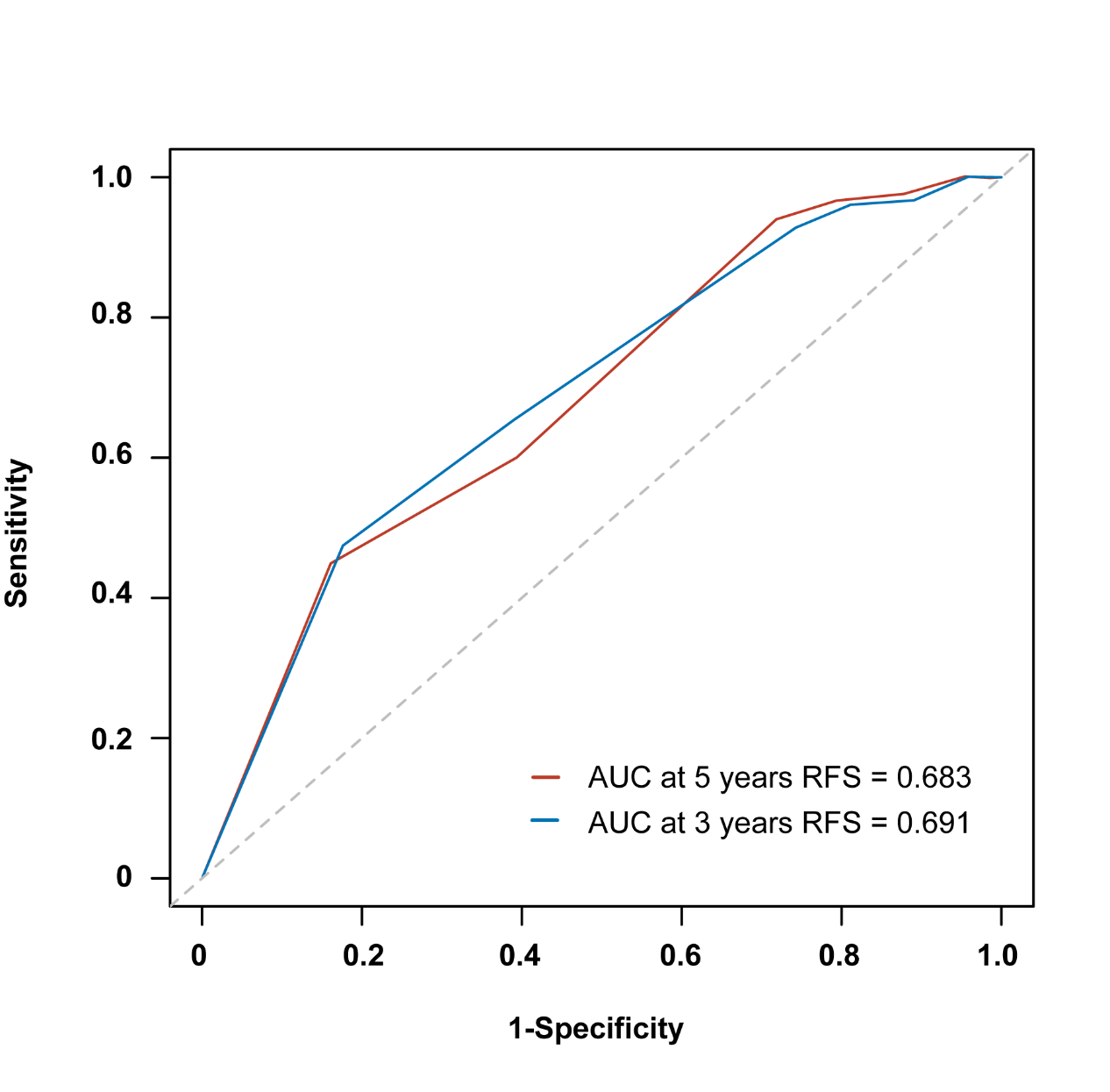


Fig. S3. Time-dependent ROC curve based on LPS from clinicopathological variables.
